# Immunophenotypic assessment of pure embryonal carcinoma and yolk sac tumour suggests that reprogramming to non‐seminoma occurs outside the spermatogonial niche

**DOI:** 10.1111/his.70160

**Published:** 2026-04-16

**Authors:** Daisy Maharjan, Květoslava Michalová, Joao Lobo, Jennifer Gordetsky, Fiona MacLean, Ankur R Sangoi, Daniel Berney, Chia‐Sui Kao, Costantino Ricci, Adeboye O Osunkoya, Maria Rosaria Raspollini, Geert JLH van Leenders, Muhammad T Idrees, Lee Ann Baldridge, Thomas M Ulbright, Andres M Acosta

**Affiliations:** ^1^ Department of Pathology Indiana University School of Medicine Indianapolis Indiana USA; ^2^ Department of Pathology Charles University, Faculty of Medicine in Plzen, Biopticka Laborator, Ltd Plzen Czech Republic; ^3^ Department of Pathology Portuguese Oncology Institute of Porto Porto Portugal; ^4^ Cancer Biology and Epigenetics Group, CI‐IPOP Porto Portugal; ^5^ ICBAS—School of Medicine and Biomedical Sciences, University of Porto Porto Portugal; ^6^ Department of Pathology and Urology Vanderbilt University Medical Center, Vanderbilt University Nashville Tennessee USA; ^7^ Douglass Hanly Moir Pathology North Ryde New South Wales Australia; ^8^ Department of Pathology Stanford Medical Center Stanford California USA; ^9^ Centre for Cancer Biomarkers & Biotherapeutics Barts Cancer Institute, Queen Mary University of London London UK; ^10^ Department of Pathology and Laboratory Medicine Cleveland Clinic Foundation Cleveland Ohio USA; ^11^ Pathology Unit, DIAP‐Dipartimento Interaziendale anatomia patologica di Bologna Maggiore Hospital‐AUSL Bologna Bologna Italy; ^12^ Department of Pathology and Laboratory Medicine Emory University School of Medicine Atlanta Georgia USA; ^13^ Department of Pathology University Hospital Careggi Florence Italy; ^14^ Department of Pathology Erasmus Medical Center Rotterdam the Netherlands

**Keywords:** embryonal carcinoma, germ cell neoplasia in situ, immunohistochemistry, yolk sac tumour

## Abstract

**Aims:**

Germ cell neoplasia in situ (GCNIS) is the precursor of GCNIS‐derived testicular germ cell tumours, including seminomas and non‐seminomas. Most non‐seminomas show mixed histology, often including components of seminoma. A small subset presents as pure non‐seminomatous germ cell tumors. In these neoplasms, the absence of invasive seminoma. raises the possibility that reprogramming to non‐seminoma may occur at an early in‐situ stage (i.e., within the spermatogonial niche). This study examined GCNIS associated with pure embryonal carcinoma (EC) and postpubertal‐type yolk sac tumour (YST) using immunohistochemistry for transcription factors characteristic of GCNIS/seminoma, EC, and YST phenotypes to determine if reprogramming to non‐seminoma occurs within the spermatogonial niche.

**Methods:**

GCNIS associated with pure testicular YST and EC was assessed using OCT4, FOXA2 (only YST), CD30 (only EC), SOX2 (only EC), SOX17, and NANOG immunohistochemistry. Adjacent foci of intratubular and invasive tumour were also evaluated, if present.

**Results:**

Sixty‐seven pure non‐seminomas (63 EC, 4 YST) were evaluated. In all cases, GCNIS expressed OCT4, SOX17, and NANOG. GCNIS associated with EC was consistently negative for CD30 and SOX2, and GCNIS associated with YST was consistently negative for FOXA2. Adjacent invasive EC (62/67 cases) and intratubular EC (19/67) showed the classic EC immunophenotype: OCT4+/NANOG+/SOX17–/SOX2+/CD30+. Similarly, invasive YST adjacent to GCNIS was OCT4–/NANOG–/SOX17+/FOXA2+.

**Conclusions:**

Assessment of transcription factors involved in the induction and maintenance of EC and YST phenotypes suggests that, in pure non‐seminomas, reprogramming occurs outside the spermatogonial niche.

## Graphical abstract

Immunohistochemical analysis of transcription factors in GCNIS associated with pure EC and YST suggests that reprogramming to non‐seminoma occurs outside the spermatogonial niche. In these tumors, reprogramming may occur immediately upon invasion of the stroma or within seminiferous tubules but outside the spermatogonial niche (i.e., in intratubular germ cell tumor components). Alternatively, these neoplasms may contain seminoma that is either unsampled or overrun by non‐seminoma at the time of orchiectomy.

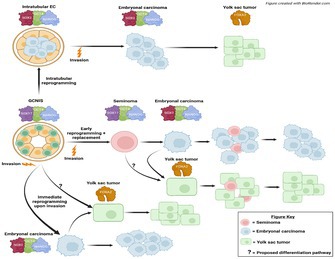

**Abbreviations**
EC: embryonal carcinoma
FFPE: formalin‐fixed, paraffin embedded
GCNIS: germ cell neoplasia in situ
NSGCTs: non‐seminomatous germ cell tumours
TGCTs: testicular germ cell tumours
YST: yolk sac tumour

## Introduction

Testicular germ cell tumours (TGCTs) represent the most common testicular neoplasms, affecting predominantly young men between 15 and 45 years of age.^1^, ^2^ Germ cell neoplasia in situ (GCNIS), the ubiquitous precursor of post‐pubertal GCNIS‐derived TGCTs, consists of neoplastic germ cells with a gonocyte‐like phenotype residing in the spermatogonial niche, at the base of the seminiferous tubules.

Primordial germ cells originate from epiblast‐derived pluripotent stem cells that migrate to the genital ridge in early embryogenesis.^3^ As they enter the spermatogonial niche during fetal and early postnatal life, they progressively commit to the germline and lose pluripotency. This commitment is marked by silencing of pluripotency markers such as OCT3/4 and NANOG, and upregulation of genes associated with the induction and maintenance of a mature spermatogonial phenotype, such as VASA, SOX17, and TSPY, changes regulated by epigenetic mechanisms including microRNA expression and DNA methylation.^4^ Failure to commit to the germline, with persistent expression of pluripotency‐associated transcription factors, is thought to be a critical step in the development of GCNIS.^5^

GCNIS‐derived TGCTs are broadly divided into seminoma and non‐seminoma for clinical management, with each subgroup comprising ~50% of these neoplasms.^2^ Non‐seminomatous germ cell tumours (NSGCTs) include embryonal carcinoma (EC), post‐pubertal‐type yolk sac tumour (YST), post‐pubertal‐type teratoma, trophoblastic tumours, and mixed neoplasms containing at least one non‐seminoma component. Among NSGCTs, most exhibit mixed histology with variable combinations of non‐seminoma and seminoma components, whereas pure tumours are rare.^6^, ^7^

Seminoma is considered the ‘default’ pathway of TGCT development and closely resembles primordial germ cells/gonocytes and GCNIS. Specifically, seminoma and GCNIS share morphologic features, epigenetic signatures, and expression of pluripotency‐associated markers such as OCT4 (POU5F1), NANOG, and SOX17.^8^, ^9^, ^10^ From a biological perspective, seminoma is hypothesized to represent a ‘latent pluripotent’ phenotype with the ability to undergo reprogramming to EC, which can further differentiate into other NSGCTs such as YST and choriocarcinoma.^11^, ^12^ It is well recognized that pure seminoma may contain admixed syncytiotrophoblast, and there is some evidence that it may occasionally undergo direct reprogramming to YST without an intermediate stage of EC.^13^, ^14^, ^15^, ^16^

Pure NSGCTs represent a challenge to the current pathogenic model, since they do not show a component of invasive seminoma. Hence, it is currently uncertain at what stage of tumorigenesis reprogramming occurs in these neoplasms. Specifically, it is not clear if reprogramming occurs in pre‐invasive GCNIS, intratubular TGCT, or an invasive seminoma component that is not represented in the histological sections. In this study, we attempted to answer this question by assessing expression of transcription factors involved in the induction and maintenance of GCNIS/seminoma, EC, and YST phenotypes in GCNIS associated with pure testicular EC and post‐pubertal type YST.

## Materials and Methods

The research was approved by the Institutional Review Board of Indiana University (protocols #18697, 2023 and #29041, 2025).

### Accrual of Study Cases

A natural language search of the Indiana University pathology database was performed to identify in‐house surgical cases diagnosed as pure EC and YST between 2016 and 2025. Slides were reviewed to identify cases containing GCNIS for assessment with immunohistochemistry. Additional pure ECs and YST cases with adjacent GCNIS were received from multiple collaborators. Representative slides of all cases were centrally reviewed at Indiana University (DM, AA) to determine includability in the study. Tumours with intratubular seminoma or minute foci of invasive seminoma that were not recognized at the time of diagnosis were excluded from the study.

### Immunohistochemistry

Immunohistochemistry was performed on formalin‐fixed, paraffin embedded (FFPE) tissue sections (4–5 μm). The samples were processed and stained following standard protocols of the Indiana University research histology laboratory. The following primary antibodies were used: anti‐OCT4 (clone MRQ10, mouse monoclonal; Cell Marque; Dilution RTU), anti‐SOX2 (clone SP76, rabbit monoclonal; Cell Marque; Dilution RTU), anti‐CD30 (clone Ber H2, mouse monoclonal; Dako Omnis; Dilution RTU), anti‐SOX17 (clone OTI2B6, mouse monoclonal; LS Bio; Dilution 1:100), anti‐NANOG (rabbit polyclonal; Cell signalling; Dilution 1:100), and anti‐FOXA2 (clone EPR4466, mouse monoclonal; Abcam; Dilution 1:500).

Expression of OCT4, SOX2, SOX17, FOXA2, and NANOG was interpreted as positive when diffuse nuclear staining was observed; weak focal staining and cytoplasmic staining for these markers was interpreted as negative. CD30 expression was interpreted as positive when diffuse membranous expression was observed; weak, focal, or non‐membranous staining was interpreted as negative. Positive controls were run for all stains.

## Results

### General Characteristics of the Study Series

**Figure 1:**
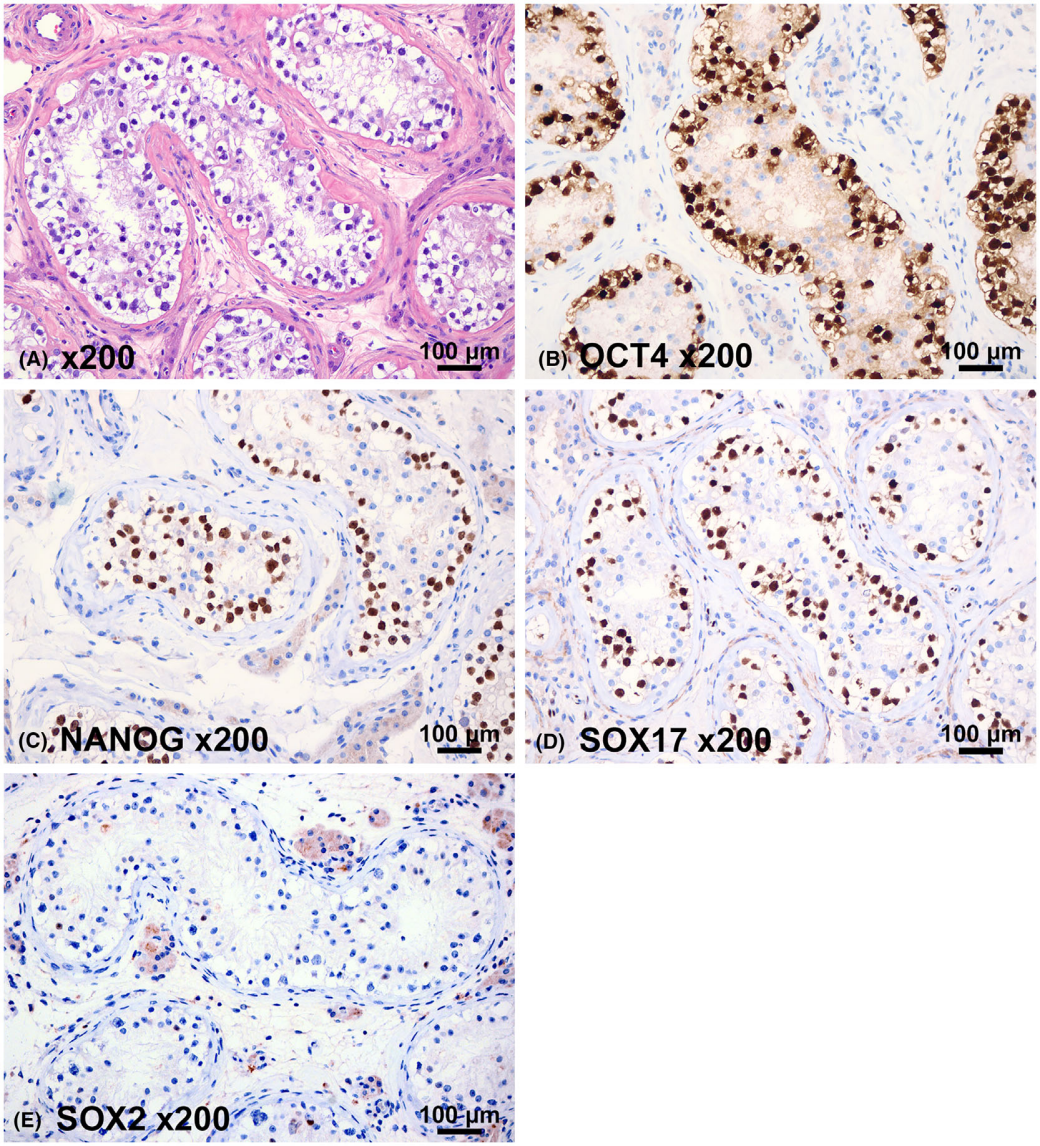
Germ cell neoplasia in situ (GCNIS) characterized by large, atypical seminoma‐like cells in the spermatogonial niche of seminiferous tubules (**A**; haematoxylin and eosin). This lesion was associated with pure embryonal carcinoma. GCNIS is positive for OCT4 (**B**), NANOG (**C**), and SOX17 (**D**). In contrast, it is negative for SOX2 (**E**).

After exclusion of non‐qualifying cases, 71 neoplasms were included in the study: 67 pure ECs and 4 pure YSTs. GCNIS was identified in the adjacent testicular parenchyma as large, atypical seminoma‐like cells located in the spermatogonial niche of the affected seminiferous tubules (Figure 1A) and confirmed with OCT4 (Figure 1B), NANOG (Figure 1C), and SOX17 (Figure 1D) immunohistochemistry.

**Figure 2:**
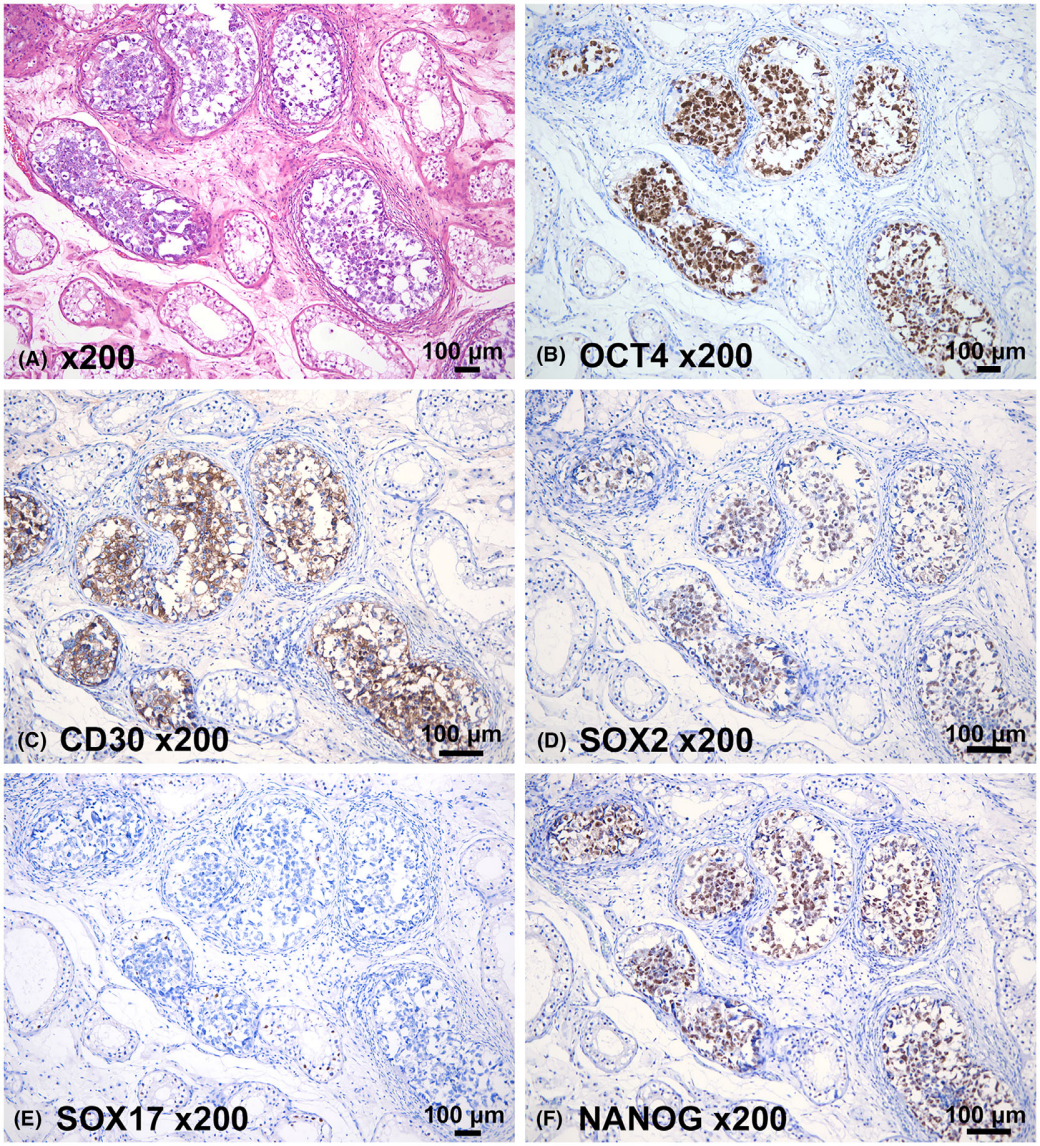
Intratubular embryonal carcinoma showing expanded tubules filled with epithelioid tumour cells with amphophilic to basophilic cytoplasm and marked nuclear atypia (**A**; haematoxylin and eosin, x200). This lesion is positive for OCT4 (**B**), CD30 (**C**), and SOX2 (**D**), while negative for SOX17 (**E**). Intratubular embryonal carcinoma is also positive for NANOG (**F**).

Among 67 cases of pure EC, invasive tumour was present adjacent to GCNIS (i.e., in the same section) in 62 samples. Intratubular components were seen in 19 ECs as expanded tubules filled with epithelioid tumour cells with amphophilic to basophilic cytoplasm and marked nuclear atypia (Figure 2A). Necrosis and calcifications were noted in the intratubular component in some cases.

**Figure 3:**
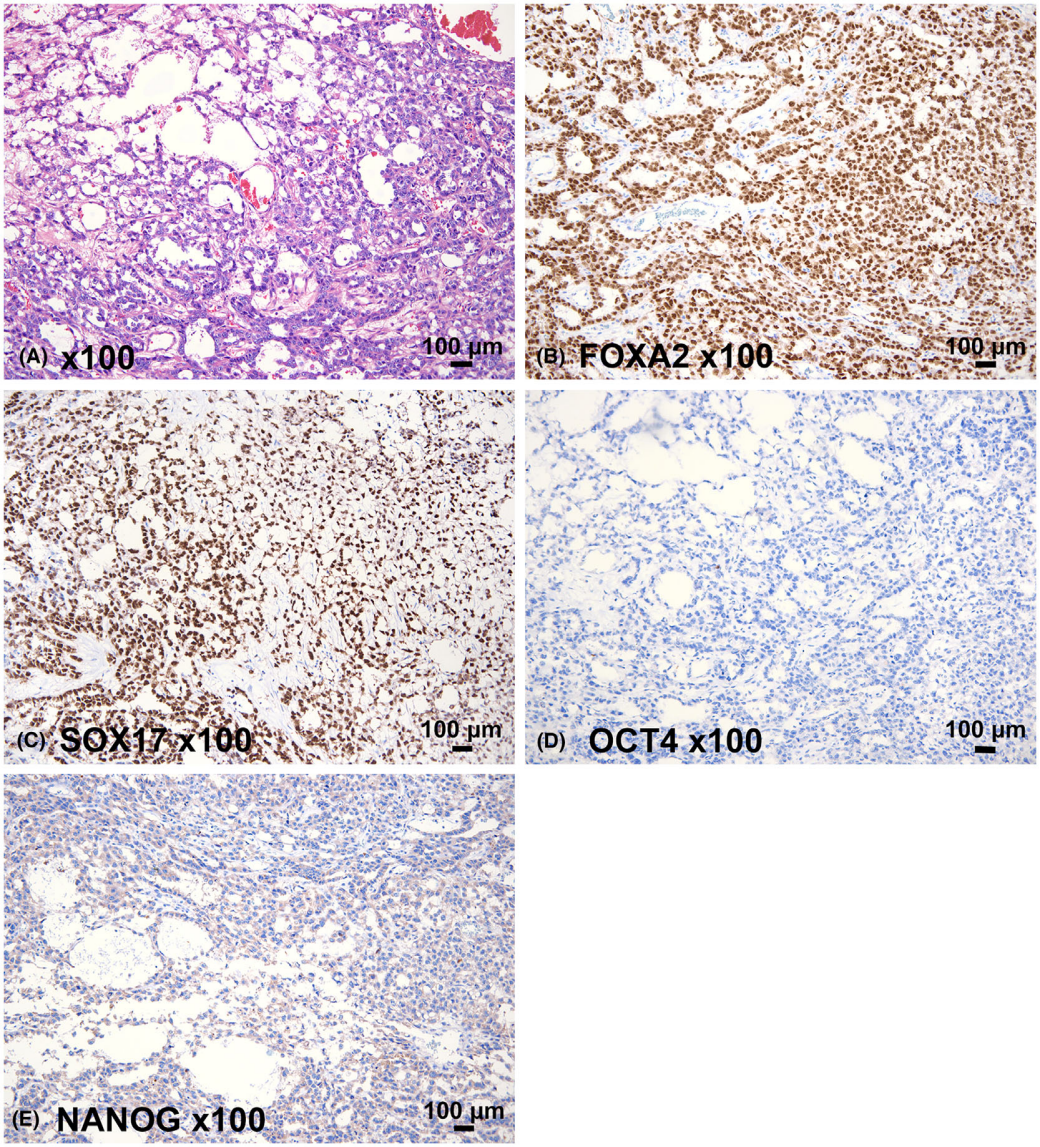
Invasive yolk sac tumour with microcystic growth pattern (**A**; haematoxylin and eosin). Yolk sac tumour is positive for FOXA2 (**B**) and SOX17 (**C**), while negative for OCT4 (**D**) and NANOG (**E**). [Colour figure can be viewed at wileyonlinelibrary.com]

Invasive components were present adjacent to GCNIS in all YSTs. The invasive components of the tumour showed microcystic, papillary, and glandular patterns (Figure 3A). No intratubular component was identified in any of these YSTs.

### Immunohistochemistry Results

Immunohistochemistry for OCT4, SOX2, CD30, SOX17, and NANOG was performed on 65/67 ECs. Among the two remaining ECs, one was assessed with OCT4, SOX2, CD30, and SOX17 and the other with OCT4, SOX2, CD30, and NANOG. The GCNIS component of these tumours was consistently positive for OCT4 (67/67), SOX17 (66/66), and NANOG (66/66) and negative for CD30 (0/67) and SOX2 (0/67). Adjacent intratubular EC components present in 19 tumours were consistently positive for OCT4 (Figure 2B), CD30 (Figure 2C), SOX2 (Figure 2D), and NANOG (Figure 2F) and negative for SOX17 (Figure 2E). Similarly, the invasive EC components of these tumours were consistently positive (62/62) for OCT4, CD30, SOX2, and NANOG and negative for SOX17 (Table 1).

**Table 1:** Immunophenotype of in situ and invasive components of pure EC and YST

	OCT4	CD30	SOX2	SOX17	NANOG	FOXA2
*Pure EC (n = 67)*						
GCNIS (*n* = 67)	67/67	0/67	0/67	66/66	66/66	0/0
Intratubular EC (*n* = 19)	19/19	19/19	19/19	0/19	19/19	0/0
Invasive EC (*n* = 62)	62/62	62/62	62/62	0/62	62/62	0/0
*Pure YST (n = 4)*						
GCNIS (*n* = 4)	4/4	0/0	0/0	4/4	4/4	0/4
Invasive YST (*n* = 4)	0/4	0/0	0/0	4/4	0/4	4/4

EC, embryonal carcinoma; GCNIS, germ cell neoplasia in situ; YST, yolk sac tumour.

Immunohistochemistry for OCT4, FOXA2, SOX17, and NANOG was performed on all (4/4) YSTs. The GCNIS component was consistently positive (4/4) for OCT4, SOX17, and NANOG and negative for FOXA2. The invasive components were consistently positive (4/4) for FOXA2 (Figure 3B) and SOX17 (Figure 3C) and negative for OCT4 (Figure 3D) and NANOG (Figure 3E) (Table 1).

## Discussion

GCNIS‐derived TGCTs, the most common solid tumours of young adult men in western countries, are considered a developmental disease. Specifically, they are the result of neoplastic transformation of primordial germ cells that fail to commit to the germline and show reversion to a pluripotent state. The underlying changes that drive tumorigenesis and reprogramming are complex, involving genomic and epigenomic alterations.^4^, ^17^ Compared with somatic tumours, GCNIS‐derived TGCTs show low mutational rates, consistent with their developmental origin.

Under the current paradigm, seminoma is regarded as a ‘latent pluripotent’ phenotype capable of reprogramming to EC and, subsequently, to other NSGCTs. Alternatively, it may undergo direct reprogramming to YST without an intervening EC intermediate. For instance, experimental studies have demonstrated that CRISPR/Cas9‐mediated depletion of SOX2 in TCam‐2 cells abolishes their capacity to reprogram into EC in vivo while preserving their ability to differentiate into YST‐like cells. These findings indicate that seminomas can undergo extra‐embryonic differentiation independent of an EC intermediate, a process in which the transcription factor FOXA2 plays a critical regulatory role.^18^, ^19^

Regardless of the pathways that seminoma follows to give rise to other histological subtypes of GCNIS‐derived TGCTs, the origin of pure NSGCTs remains somewhat enigmatic. More specifically, these tumours lack an identifiable component of invasive seminoma, the proposed invasive precursor of all NSGCT subtypes. Therefore, it is hypothetically possible that, in pure NSGCTs, reprogramming occurs at a pre‐invasive stage (i.e., within the spermatogonial niche). To explore this hypothesis, we used immunohistochemistry for transcription factors identified as key regulators of the GCNIS/seminoma, EC, and YST phenotypes to assess GCNIS associated with pure testicular EC and YST.

SOX17 is a transcription factor that interacts with OCT4 in GCNIS and seminoma to regulate *TFAP2C*, *PRDM1*, and *PRDM14*, a gene expression programme of latent pluripotency that inhibits somatic differentiation.^20^, ^21^ Reprogramming of seminoma to EC is marked by suppression of SOX17 and expression of SOX2, which pairs with OCT4 to regulate EC‐specific transcription programmes.^17^, ^20^ This results in distinct immunoprofiles, with seminoma being NANOG+/OCT4+/SOX17+/SOX2‐/CD30‐ and EC being NANOG+/OCT4+/SOX17‐/SOX2+/CD30+ .^10^, ^22^, ^23^ Differentiation of EC to YST is marked by downregulation of the pluripotency markers NANOG/OCT4 and SOX2 with concurrent upregulation of FOXA2 and reactivation of SOX17, with progressive acquisition of cisplatin resistance.^24^ FOXA2 has been identified as a central molecular driver of YST development, inducing expression of ‘canonical’ YST markers such as AFP, GPC3, APOA1/APOB, ALB, and transcription factors of the GATA family.^25^ Upregulation of FOXA2 also shifts SOX17 from a pluripotency‐promoting factor to a differentiation‐inducing factor.^25^ Consistently, YST is characterized by a NANOG‐/OCT4‐/SOX17+/FOXA2+ immunophenotype.^25^, ^26^

If reprogramming to NSGCT in pure EC and YST occurred at an early pre‐invasive stage (i.e., in GCNIS), neoplastic germ cells mirroring the immunoprofile of these tumour types should be present in the spermatogonial niche. Our results show that GCNIS components of pure EC and YST invariably exhibit the typical NANOG+/OCT4+/SOX17+/SOX2‐/CD30‐ immunophenotype of GCNIS/seminoma, whereas adjacent invasive EC and YST is consistently NANOG+/OCT4+/SOX17‐/SOX2+/CD30+ and NANOG‐/OCT4‐/SOX17+/FOXA2+, respectively. This suggests that, in line with current assumptions, GCNIS does not undergo direct reprogramming to non‐seminoma. The presence of intratubular EC with an immunoprofile identical to that of adjacent invasive EC raises the possibility that reprogramming may occur within seminiferous tubules after the neoplastic germ cells leave the spermatogonial niche. However, it is currently uncertain whether intratubular EC represents a pre‐invasive lesion or retrograde spread of invasive tumour into seminiferous tubules.

**Figure 4:**
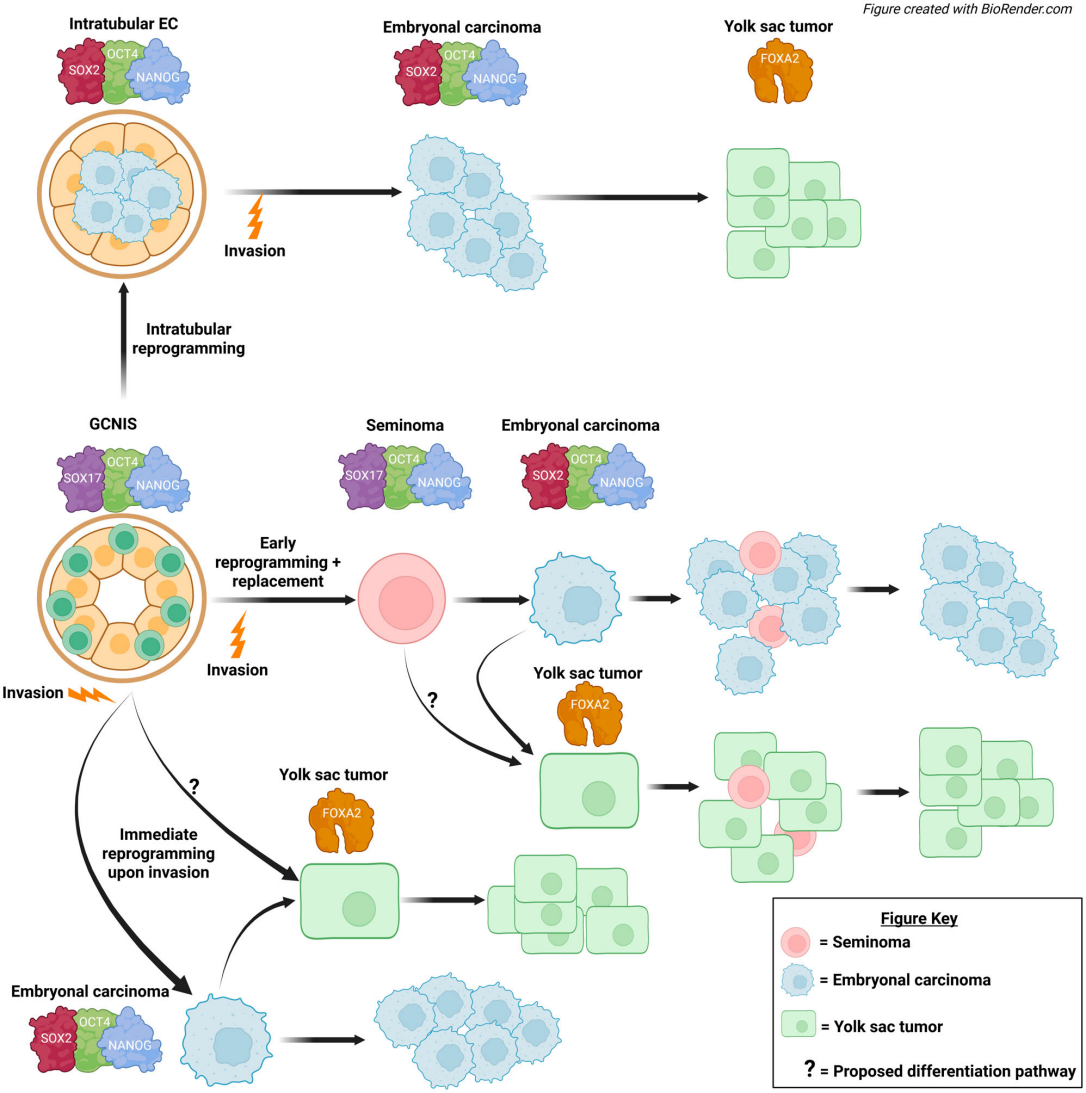
Possible pathways of reprogramming in pure non‐seminomatous germ cell tumours. [Colour figure can be viewed at wileyonlinelibrary.com]

Our study shows that, in pure NSGCTs, reprogramming occurs outside the spermatogonial niche. Hence, the remaining hypothetical possibilities are that reprogramming occurs (1) immediately upon invasion of the intertubular stroma (i.e., without an intermediate stage of seminoma); (2) within seminiferous tubules after the neoplastic germ cells leave the spermatogonial niche (i.e., in intratubular germ cell tumor); or (3) via an intermediate stage of seminoma that is overrun by NSGCT, being no longer present or not captured by representative sampling at the time of orchiectomy (Figure 4). Although these scenarios are not mutually exclusive, we believe that the latter is most likely. This conclusion is supported in part by the absence of intratubular components in most ECs and YSTs analysed herein. Studies using the seminoma‐derived TCam‐2 cell line and clinical samples have demonstrated that the tumour microenvironment, including tumour‐associated inflammatory cells present in the stroma, may have a central role in regulating the reprogramming of seminoma to NSGCTs.^13^, ^18^, ^19^, ^27^, ^28^, ^29^, ^30^, ^31^ Our results support that a change in the microenvironment is likely needed for reprogramming to occur. Further studies are needed to properly delineate the biological processes that induce the development of NSGCTs.

## Author contributions

AMA conceptualized and coordinated the research. AMA and DM assessed the cases (including immunohistochemistry) and drafted the manuscript. LAB performed the immunohistochemistry and DM collected the data. All authors made intellectual and/or material contributions to the study and read/edited the manuscript.

## Funding Statement

The authors have nothing to report.

## Conflict of Interest

The authors declare that they have no conflicts of interest pertaining to the contents of this manuscript.

## Data Availability

The data generated in this study is available from the corresponding author upon reasonable request.
